# The *in vitro* effects of acidemia and acidemia reversal on coagulation in dogs

**DOI:** 10.3389/fvets.2024.1427237

**Published:** 2024-09-05

**Authors:** Youngju Kim, Hyeona Bae, DoHyeon Yu

**Affiliations:** College of Veterinary Medicine, Gyeongsang National University, Jinju, Republic of Korea

**Keywords:** acidosis, dog, hemostasis, blood coagulation factors, thromboelastography

## Abstract

**Background:**

The effect of acidemia on blood coagulation remains inadequately understood in veterinary medicine. Therefore, we assessed the effect of *in vitro* acidification of canine whole blood on coagulation and investigated whether acidemia-induced coagulopathy could be reversed by reversing acidemia.

**Methods:**

Citrated whole blood samples were taken from six healthy Beagle dogs and categorized, based on pH adjustment, into neutral, weak acidemia (WA), strong acidemia (SA), and reversal from SA. Then, prothrombin time (PT), activated partial thromboplastin time (aPTT), fibrinogen concentration, conventional thromboelastography (TEG) parameters, and velocity curve (V-curve) variables of TEG were assessed.

**Results:**

The PT, aPTT, and most TEG parameters showed significant coagulopathy in the SA group compared to the neutral group, with additional significant changes in reaction time (R), clot kinetic (K), maximum amplitude (MA), split point (SP), elasticity (E), thrombodynamic potential index (TPI), and coagulation index (CI) between the SA and WA groups. Among V-curve variables, the maximum rate of thrombus generation (MRTG) and total thrombus generation were significantly inhibited in the SA group compared to the neutral group, with significant differences in the time to maximum rate of thrombus generation (TMRTG) between the WA and SA groups. In the reverse group, aPTT, R, K, α-angle, MRTG, TMRTG, SP, TPI, and CI exhibited significant recovery compared to the SA group.

**Conclusion:**

The *in vitro* induction of acidemia in canine whole blood leads to impairment of coagulation profiles, and pH correction can reverse most acidemia-induced coagulopathy.

## 1 Introduction

Blood pH is tightly regulated within a narrow range (pH: 7.35–7.45) and deviations from this range can have serious health implications. Acidemia is commonly observed in critically ill patients with metabolic derangements or trauma, and it also plays a significant role in veterinary medicine, influencing various animal diseases and pathophysiological conditions such as respiratory diseases (e.g., pneumonia, asthma), metabolic disorders like diabetic ketoacidosis and renal failure, hypoxia-related conditions such as septic shock and heart failure, poisoning from substances like ethylene glycol or salicylates, liver failure, and pregnancy toxemia in ruminants. Acidemia has several adverse effects including decreased cardiac contractility, ventricular arrhythmia, arterial vasodilation, and catecholamine resistance (1–4). Ultimately, acidemia can influence the body’s ability to form and maintain blood clots, resulting in coagulopathy. This association is evident in the well-known “lethal triad” of hypothermia, acidosis, and coagulopathy, which has been linked to increased mortality after major trauma (5–8).

Previous *in vitro* human studies demonstrated a strong correlation between pH levels and coagulopathy in blood samples infused with acid, typically hydrochloric acid (HCl), from healthy volunteers (9, 10). *In vivo* studies of swine models found that HCl-induced acidemia leads to coagulopathy, as measured by prothrombin time (PT), activated partial thromboplastin time (aPTT), fibrinogen concentration, conventional thromboelastography (TEG) parameters, and thrombin generation tests (11, 12). However, research on the effects of acidemia on coagulation is limited in veterinary medicine, and the results are often inconsistent (13–15).

Considering the lack of research in this field on dogs, the primary objective of this study was to investigate the effects of *in vitro* acidification of canine whole blood on coagulation to identify the correlation between acidemia and coagulopathy. Furthermore, this study aimed to investigate whether acidemia-induced coagulopathy could be reversed by reversing acidemia.

## 2 Materials and methods

### 2.1 Study design

This study used an *in vitro* assay to evaluate the effects of acidification of canine whole blood on coagulation and investigate the potential recovery of these effects upon reversing acidemia. Blood samples were categorized into four groups: weak acidemia (WA), strong acidemia (SA), neutral, and reverse. The acidification group was further divided into WA and SA groups to assess progressive coagulation impairment with worsening acidemia (15, 16). The research protocol was reviewed and approved by the Institutional Animal Care and Use Committee (IACUC) GNU-231017-D0193 of Gyeongsang National University (GNU).

#### 2.1.1 Blood sample collection

Six Beagle dogs consisting of four castrated males and two spayed females with median body weight and age of 10.8 ± 1.3 kg and 7.0 ± 1.4 years, respectively, were included in this study. The dogs were deemed healthy based on physical examination, complete blood count (Procyte Dx Hematology Analyzer; IDEXX, Westbrook, ME, United States), serum biochemistry analysis (Catalyst Dx^®^ Chemistry Analyzer; IDEXX, Westbrook, ME, United States), acid–base balance and electrolyte concentration analysis, and coagulation profiles.

Blood samples were collected from each dog using the two-syringe method with a 21 G butterfly needle, following the recommended guideline to minimize stasis for TEG analysis (17). The procedure involved discarding the initial 3 mL of blood drawn from the jugular vein and collecting 10 mL of blood from each dog. The 10 mL blood samples were transferred into five sodium citrate 2-mL tubes, each containing 0.2 mL of buffered 3.2% sodium citrate, resulting in a volume ratio of 1:9 (Vacuette, 3.2% sodium citrate, 2-mL tubes; Greiner Bio-One, Kremsmünster, Austria). Subsequently, the citrated blood tubes were gently inverted 5–7 times to ensure that the ratio of sodium citrate to whole blood was maintained at 1:9. Four tubes were used for the neutral, WA, SA, and reverse groups, and one tube was used for pH adjustment.

#### 2.1.2 Blood sample acidification and reversal

The blood samples in the acidification group were all acidified using the same 1 M HCl (Sigma-Aldrich, St. Louis, MO, United States) solution. The pH change was measured after adding 10 μL of 1 M HCl to the blood sample for pH adjustment. Subsequently, 1 M HCl was added to the other two citrate tubes to obtain the WA and SA samples (target pH: 6.8–7.0 and 6.5–6.6, respectively). The median volume of HCl administered for acidemia induction was 30 μL (range, 25.0–32.5 μL) and 59.5 μL (range, 51–60 μL) in the WA and SA groups, respectively. The fourth blood sample was used as the neutral group, and an amount of distilled water similar to that of the HCl administered to induce SA was added to eliminate the dilutional effect. Strong acidemia was induced and then reversed by adding 45 μL of Tris (hydroxymethyl) aminomethane buffer (1.5 M Tris–HCl, pH, 8.8, Bio-Solution co., LTD., Suwon, Korea) for the reverse group. Tris buffer was used instead of sodium bicarbonate for acidemia reversal because CO_2_ is generated when bicarbonate neutralizes protons (H^+^), which is unsuitable for *in vitro* experiments conducted in closed systems.

The pH of the blood samples was confirmed using the blood gas analyzer (Stat Profile^®^ pHOX Ultra Analyzer; Nova Biochemical, Waltham, MA, United States) immediately after manipulation.

### 2.2 Analysis of coagulation profiles

Point-of-care PT, aPTT, fibrinogen concentration, and TEG were analyzed within 2 h of blood collection using the citrated whole blood.

#### 2.2.1 TEG

The samples were analyzed using a single TEG^®^ 5,000 Thromboelastograph Hemostasis Analyzer (Haemonetics Corp, Braintree, Massachusetts, United States). TEG is a non-invasive test that quantitatively measures the ability of whole blood to form a clot. The test detects and quantifies dynamic changes in the viscoelastic properties of blood during clotting under low shear stress. Citrated whole blood samples were analyzed after a 30-min resting period at room temperature, following a previously recommended protocol (17). All the samples were activated using kaolin, in accordance with the manufacturer’s recommendations.

**Figure 1:**
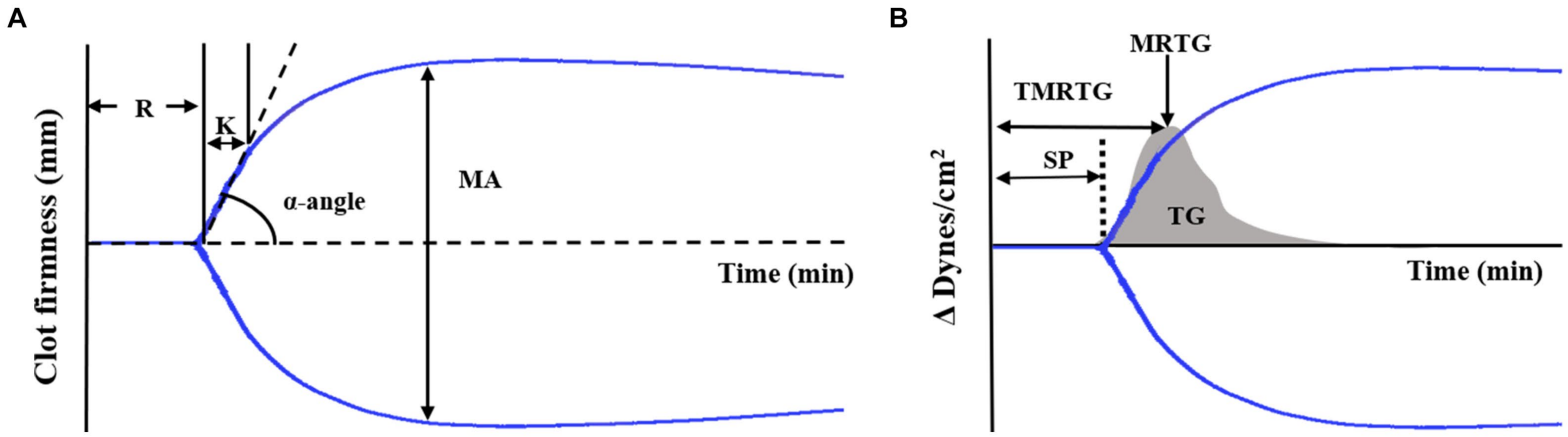
TEG tracing and velocity (first derivative) curve **(A)** Standard TEG tracing. The four conventional coagulation parameters (R, K, α-angle, MA) can be combined to yield indices of coagulability. **(B)** TEG showing the standard tracing (black line) with the superimposed velocity curve (gray solid curve). Velocity curve parameters are generated from the mathematical first derivative of the standard TEG tracing and describe the formation of the thrombus. Thromboelastography (TEG), reaction time (R), clot kinetic (K), α-angle, maximum amplitude (MA), split point (SP), maximum rate of thrombus generation (MRTG), time to maximum rate of thrombus generation (TMRTG), and total thrombus generation (TG).

Regarding TEG conventional parameters, the reaction time (R) represents initial clot latency, the clot kinetic (K) is the duration from the initial to maximum clot formation, the α-angle measures the rapidity of fibrin buildup and cross-linking, and the maximum amplitude (MA) represents the maximum strength of the clot (Figure 1A).

Additionally, the TEG system software calculated the first-degree derivative velocity curve (V-curve), providing information about thrombin generation (Figure 1B). The MRTG corresponds to the peak of the clot formation curve and the time to maximum rate of thrombus generation (TMRTG) is determined by measuring the time it takes for the clot to reach its peak formation rate. Moreover, TG is calculated by assessing the area under the thrombus formation rate curve (18).

Furthermore, several non-conventional TEG parameters were analyzed. These parameters included split point (SP, the first point at which the arms of the thromboelastogram diverge) (Figure 1B), amplitude (A, equal to MA until MA is determined), shear elastic modulus strength (G, clot elasticity at the specific time of reaching the maximal amplitude, calculated from A as follows: G = 5,000A/[100-A]), elasticity (E, a normalized G parameter, calculated as E = 100A/[100-A]), thrombodynamic potential index (TPI, describing the global coagulation, calculated as SP/R + K), coagulation index (CI, describing overall coagulation, calculated as 0.1227 × R + 0.0092 × K + 0.1654 × MA − 0.0241 × Angle−5.0220), and time to maximum amplitude (TMA, the time needed to form a stable clot).

#### 2.2.2 Point-of-care PT and aPTT

After the TEG analysis, PT and aPTT were measured using a point-of-care device and cartridges (Coag Dx™ Analyzer, Citrate PT, and Citrate aPTT cartridges; IDEXX, Westbrook, ME, United States). The measurements were conducted using the same citrate tube used for the TEG analysis, following the manufacturer’s guidelines.

#### 2.2.3 Fibrinogen concentration

The remaining citrated blood was centrifuged at 2000 × g for 15 min, and fibrinogen analysis was conducted using the supernatant of the citrated plasma. Fibrinogen concentration was determined using the STA-Compact-Max^®^ analyzer (Diagnostica Stago, Asnières sur Seine, France), following the manufacturer’s instructions.

### 2.3 Statistical methods

All statistical analyses were performed using SPSS for Windows (version 27.0; SPSS Inc., Chicago, IL, United States) with nonparametric statistical tests. Statistical analysis was performed using the initial Kruskal–Wallis test, followed by the post-hoc Mann–Whitney U test with Bonferroni correction to compare the neutral, WA, and SA variables. The Mann–Whitney U test was used to compare the two groups (SA and reverse). The Spearman rank order test was used for correlation analysis between pH and coagulation impairment. The results are provided as median (range), represented using box plots, with boxes representing the 25–75th percentiles and whiskers representing the minimum and maximum values. Statistical significance was set at *p* < 0.05. All graphical analyses were performed using the GraphPad Prism 5 software (GraphPad Software, La Jolla, CA, United States).

## 3 Results

The median pH was 7.25 (range, 7.22–7.28), 6.87 (range, 6.82–6.91), and 6.57 (range, 6.51–6.58) for the neutral, WA, and SA groups, respectively. Severe acidemia was successfully corrected with Tris buffer for the reverse group, resulting in a median pH of 7.26 (range, 7.14–7.28) (Table 1).

**Table 1:** Effect of acidification and reversal on pH level, PT, aPTT, and fibrinogen concentration for each group.

Group	pH	PT (sec)	aPTT (sec)	Fibrinogen (mg/dl)
Neutral	7.25 (7.22–7.28)	14.0 (12.0–15.5)	85.0 (76.0–90.7)	172.5 (143.0–206.2)
WA	6.87* (6.82–6.91)	16 (14.0–17.5)	93.5 (81.0–100.7)	208.0 (173.0–222.7)
SA	6.57*§ (6.51–6.58)	18.5* (16.0–21.2)	117.5*§ (104.0–133.0)	186.5 (154.0–220.2)
Reverse	7.26† (7.14–7.28)	16.5 (14.0–18.5)	97.0† (90.0–106.2)	169 (137.0–179.2)

Data are presented as median (range). Prothrombin time (PT), activated partial thromboplastin time (aPTT).*, *p* < 0.05 versus neutral group; §, *p* < 0.05 versus weak acidemia (WA) group; †, *p* < 0.05 versus strong acidemia (SA) group.

### 3.1 Effect of acidification and reversal on PT, aPTT, and fibrinogen concentration

**Figure 2:**
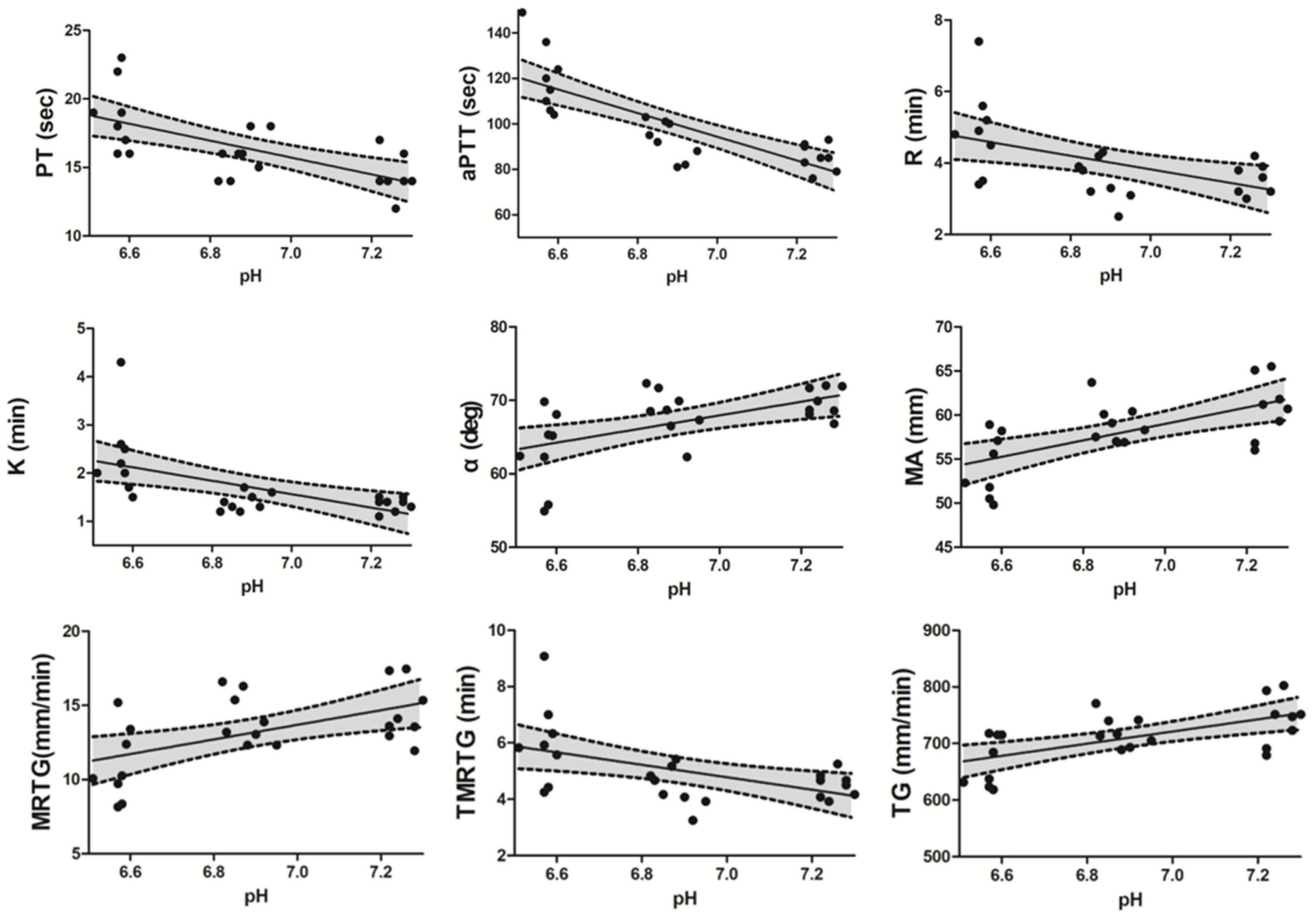
Spearman correlations between PT, aPTT, conventional TEG parameters (R, K, α-angle, and MA), and pH level, as well as between TEG velocity curve parameters and pH level. A negative correlation was observed between pH level and PT (*r* = −0.663, *p* < 0.001), aPTT (*r* = −0.866, *p* < 0.001), R (*r* = −0.533, *p* = 0.007), K (*r* = −0.645, *p* < 0.001), and TMRTG (*r* = −0.530, *p* = 0.008) within the pH range 6.5–7.3, whereas a positive correlation was observed between pH level and α-angle (*r* = 0.514, *p* = 0.01), MA (*r* = 0.638, *p* = 0.001), MRTG (*r* = 0.472, *p* = 0.02) as well as TG (*r* = 0.603, *p* = 0.002). A 95% confidence interval (CI) is reported in each scatter plot. Prothrombin time (PT), activated partial thromboplastin time (aPTT), Thromboelastography (TEG), reaction time (R), clot kinetic (K), α-angle, maximum amplitude (MA), maximum rate of thrombus generation (MRTG), time to maximum rate of thrombus generation (TMRTG), and total thrombus generation (TG).

The PT (*p* = 0.001) and aPTT (*p* < 0.001) tests showed significant prolongation in the SA group compared to the neutral group. Additionally, a significant prolongation was observed in aPTT in the SA group compared to the WA group (*p* < 0.001) (Table 1). A negative correlation between pH value and PT (*r* = −0.663, *p* < 0.001) as well as aPTT (*r* = −0.866, *p* < 0.001) were observed within the pH range 7.3–6.5 (Figure 2). A significant recovery of aPTT was observed in the reverse group compared to the SA group (*p* = 0.002) upon acidemia reversal. Furthermore, PT showed a tendency to recover in response to acidemia reversal compared to the SA group; however, statistical significance was not achieved (Table 1). No significant change was observed in fibrinogen concentration (Table 1).

### 3.2 Effect of acidification and reversal on TEG parameters

Significant differences were identified in conventional TEG parameters, including K (*p* < 0.001), α-angle (*p* = 0.003), and MA (*p* = 0.005) between the neutral and SA groups (Table 2). Additionally, significant changes were observed in R (*p* = 0.01), K (*p* = 0.001), and MA (*p* = 0.015) in the SA group compared to the WA group (Table 2). A negative correlation was observed between pH value and R (*r* = −0.533, *p* = 0.007) as well as K (*r* = −0.645, *p* < 0.001) within the pH range 7.3–6.5. A positive correlation was observed between pH value and α-angle (*r* = 0.514, *p* = 0.01) as well as MA (*r* = 0.638, *p* < 0.001) (Figure 2). Significant recovery of R (*p* = 0.001), K (*p* < 0.001), and α-angle (*p* < 0.001) was observed in the reverse group compared to the SA group. Moreover, in the reverse group compared with the SA group, MA increased without significant difference (Table 2).

**Table 2:** Effect of acidification and reversal on TEG conventional parameters and TEG velocity curve parameters for each group.

Group	R (min)	K (min)	α-angle (deg)	MA (mm)	MRTG (mm/min)	TMRTG (min)	TG (mm/min)
Neutral	3.7 (3.0–3.8)	1.4 (1.1–1.4)	69.3 (66.8–71.8)	60.9 (56.0–64.2)	3.7 (3.0–3.8)	1.4 (1.1–1.4)	69.3 (66.8–71.8)
WA	3.5 (2.5–4.1)	1.3 (1.2–1.5)	68.6 (62.3–71.2)	58.7 (56.9–60.3)	3.5 (2.5–4.1)	1.3 (1.2–1.5)	68.6 (62.3–71.2)
SA	4.8§ (3.4–5.5)	2.1*§ (1.5–2.5)	63.8* (54.9–67.4)	53.9*§ (49.8–57.9)	4.8§ (3.4–5.5)	2.1*§ (1.5–2.5)	63.8* (54.9–67.4)
Reverse	3.1† (2.8–3.4)	1.4† (1.2–1.4)	71.1† (68.5–72.1)	58.1 (56.5–60.7)	3.1† (2.8–3.4)	1.4† (1.2–1.4)	71.1† (68.5–72.1)

Data are presented as median (range). Reaction time (R), clot kinetic (K), maximum amplitude (MA), maximum rate of thrombus generation (MRTG), time to maximum rate of thrombus generation (TMRTG), and total thrombus generation (TG).*, *p* < 0.05 versus neutral group; §, *p* < 0.05 versus weak acidemia (WA) group; †, *p* < 0.05 versus. Strong acidemia (SA) group.

In the SA group compared with the neutral group, MRTG (*p* = 0.01) and TG (*p* = 0.007) were significantly inhibited (Table 2). Moreover, a significant difference in the TMRTG (*p* = 0.01) was identified between the WA and SA groups (Table 2). A positive correlation was observed between pH value and MRTG (*r* = 0.472, *p* = 0.02) as well as TG (*r* = 0.603, *p* = 0.002), and a negative correlation was observed between pH and TMRTG (*r* = −0.530, *p* = 0.008) (Figure 2). Furthermore, MRTG (*p* = 0.01) and TMRTG (*p* = 0.001) significantly recovered in the reverse group compared to the SA group, whereas TG increased with pH correction, although non-significantly (Table 2).

**Figure 3:**
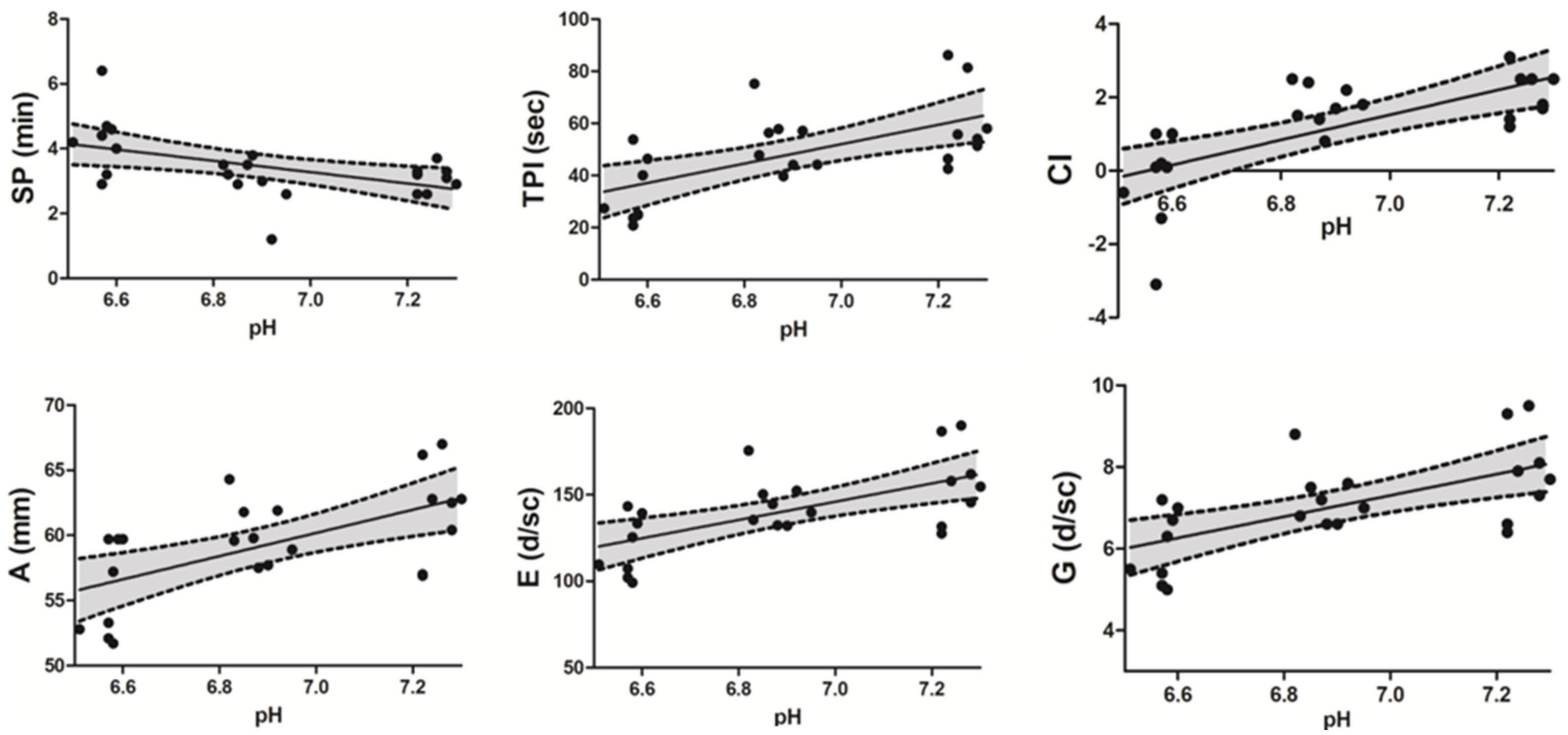
Spearman correlations between nonconventional TEG parameters and pH level. A negative correlation was observed between pH level and SP (*r* = −0.534, *p* = 0.007) within the pH range 6.5–7.3, whereas a positive correlation was observed between pH level and G (*r* = 0.636, *p* < 0.001), E (*r* = 0.638, *p* < 0.001), TPI (*r* = 0.60, *p* = 0.002), A (*r* = 0.602, *p* = 0.002), and CI (*r* = 0.744, *p* < 0.001). A 95% confidence interval (CI) is reported in each scatter plot. Thromboelastography (TEG), split point (SP), thrombodynamic potential index (TPI), coagulation index (CI), amplitude (A), elasticity or elastic modulus (E), Shear Elastic Modulus (G).

Nonconventional TEG parameters showed significant differences in SP, G, E, TPI, A, and CI, except TMA, between the neutral and SA groups (Table 3). Additionally, SP (*p* = 0.015), E (*p* = 0.015), TPI (*p* = 0.01), and CI (*p* = 0.01) showed significant differences between the WA and SA groups (Table 3). A negative correlation was observed between the pH value and SP (*r* = −0.4534, *p* = 0.007) within the pH range 7.3–6.5, whereas a positive correlation was observed between the pH value and G (*r* = 0.636, *p* < 0.001), E (*r* = 0.638, *p* < 0.001), TPI (*r* = 0.60, *p* = 0.002), A (*r* = 0.602, *p* = 0.002) and CI (*r* = 0.744, *p* < 0.001) (Figure 3). Additionally, SP (*p* = 0.003), TPI (*p* = 0.01), and CI (*p* < 0.001) showed significant recovery between the reverse and SA groups. Similarly, G, E, and A values increased compared to those in the SA group, although non-significantly (Table 3).

**Table 3:** Effect of acidification and reversal on TEG nonconventional parameters for each group.

Group	SP (min)	TMA (min)	G (d/s)	E (d/s)	TPI (sec)	A (mm)	CI
Neutral	3.1 (2.6–3.3)	24.5 (22.2–25.3)	7.8 (6.4–9.0)	156.3 (127.5–180.5)	54.8 (42.5–75.5)	62.6 (56.9–36.3)	2.1 (1.2–2.5)
WA	3.1 (1.2–3.5)	22.9 (21.4–24.4)	7.1 (6.6–7.5)	142.2 (132.0–151.8)	52.1 (39.7–57.6)	59.7 (57.5–61.8)	1.7 (0.8––2.3)
SA	4.3*§ (2.9–4.6)	25.5 (22.2–26.2)	5.9* (5.0–6.9)	117.5*§ (99.2–137.8)	26.2*§ (20.8–44.8)	55.2* (51.7–59.7)	0.1*§ (−3.1–0.8)
Reverse	2.8† (2.6–3.1)	25.7 (24.3–27.4)	6.9 (6.5–7.7)	138.6 (130.1–154.7)	48.0† (43.4–54.6)	58.8 (57.7–61.3)	2.1† (1.1–2.5)

Data are presented as median (range). Split point (SP), time to maximum amplitude (TMA), Shear Elastic Modulus (G), elasticity or elastic modulus (E), thrombodynamic potential index (TPI), amplitude (A), and coagulation index (CI).*, *p* < 0.05 versus neutral group; §, *p* < 0.05 versus weak acidemia (WA) group; †, *p* < 0.05 versus. Strong acidemia (SA) group.

## 4 Discussion

This study investigated the effects of acidemia on coagulation in dogs using several coagulation profiles and the recovery from acidemia-induced coagulopathy after reversing acidemia. The *in vitro* induction of acidemia from pH 7.28 to 6.51 in canine whole blood significantly impaired most coagulation parameters, and a correlation was observed between pH and these coagulation impairments, except for fibrinogen and TMA levels. Most coagulation profiles exhibited significant recovery upon acidemia reversal.

Acidemia significantly and progressively prolonged PT and aPTT as the pH decreased. In general, PT and aPTT are used to evaluate clotting ability, and significant changes in them indicate the inhibition of overall coagulation factor activity (19–23). Lowering the pH value of whole blood to 7.0 decreased FVIIa activity by >90% and FVIIa/TF activity by >60%, and the rate of prothrombin activation by FXa/Fva complexes by approximately 70% (24). Previous *in vivo* swine studies demonstrated that PT and aPTT were prolonged when acidemia was induced (25, 26). Consequently, acidemia significantly affects the coagulation process by interfering with the activation of coagulation factors.

Thrombin generation is a crucial step in blood clot formation (27, 28). This process involves a series of complex biochemical reactions that ultimately lead to the conversion of fibrinogen into fibrin strands (29, 30). The thrombus velocity curve derived from conventional TEG parameters was first described by Sorensen et al. and showed a “profile similarity” to thrombin generation curves (31). Rivard et al. revealed a strong correlation between TG—derived from the first derivative of the TEG waveform—and thrombin generation—assessed using the thrombin/antithrombin (TAT) complex—indicating the potential utility of the V-curve as a surrogate marker for thrombin generation (32). In this study, MRTG, TMRTG, and TG were inhibited by severe acidemia, and coagulation impairment progressively worsened with decreasing pH. A study examining changes in thrombin generation kinetics under acidic conditions using swine found that initial thrombin generation at pH 7.1 was moderately delayed. After initial thrombin formation, acidemia drastically inhibited the thrombin generation rate during the propagation phase (33). Therefore, the inhibition of TEG V-curve variables observed in this experiment indicates that acidemia suppresses thrombin generation.

Severe acidemia affected other TEG parameters. Prolonged R time indicated delayed fibrin clot formation due to acidemia, reflecting the inhibition of coagulation factor activation. Moreover, K time and α-angle are primarily influenced by fibrinogen levels and, to a lesser extent, platelet function. Fibrinogen is a crucial protein in the blood clotting process and acts as a precursor to fibrin. Previous *in vivo* swine experiments demonstrated a significant decrease in fibrinogen concentration during acidemia (11, 12) and recent research has revealed that acidemia increases fibrinogen degradation by 1.8-fold (25). However, unlike the results of previous studies, this study did not confirm any change in fibrinogen concentration with acidemia. The reason for the lack of significant changes in fibrinogen concentration is unclear but may be attributed to the *in vitro* design, which cannot reproduce the fibrinogen degradation process caused by the complex and diverse factors in living organisms. Nevertheless, K and α-angle, which are significantly influenced by fibrinogen, were notably impaired in this experiment, leading to the assumption that this may have resulted from the acidemia-induced fibrinogen dysfunction.

The MA and G values significantly decreased with acidemia, indicating that acidemia weakens blood clot strength. The inhibition of platelet function may have played a significant role in these changes since no significant changes in fibrinogen levels were observed; this is supported by previous studies showing that platelet function is suppressed in acidic environments (34). Another study measuring platelet aggregation in canine blood found that increasing acidemia led to a mild but significant decrease in platelet aggregation (35). Although TEG parameters do not independently assess platelet function, the collective findings of these studies support the conclusion that acidemia affects platelet function. However, this may have been partially influenced by fibrinogen dysfunction, as previously described.

Furthermore, significant impairment was observed in nonconventional TEG parameters in severe acidemia. This impairment progressively increased as the pH decreased. The significant impairment in these parameters was likely caused by the inhibition of coagulation factor activation, thrombin generation, and platelet function since these parameters are calculated from conventional TEG (R, K, Angle, and MA) and G parameters.

This experiment demonstrated impairment in most coagulation parameters in severe acidemia; however, the results of some previous *in vitro* studies of humans and dogs showed that acidemia alone resulted in mild or no coagulation impairment (15, 36–38). A study assessing the effects of an acidic environment on coagulation dynamics using synthetic plasma reported that the rate constants for antithrombin (AT) inhibition were reduced by approximately 25–30% at pH 7.0 compared to pH 7.4. This suggests that the decreased reactivity of AT, which is suppressed in acidic environments, toward coagulation proteases may counteract the impairment of procoagulant catalysis caused by acidification. Accordingly, the severity of acidemia and features of *in vitro* experiments may have influenced the results. Acidemia was induced at levels similar to those in our WA group in previous *in vitro* experiments in which acidemia alone could not confirm apparent coagulation failure (15, 36–38). Similar to previous results, no statistically significant coagulation failure was confirmed in the WA group in this experiment compared to the neutral group. Therefore, the lack of apparent coagulation failure can be explained by the AT inhibition effect at pH levels similar to those in our WA group. However, the hypocoagulable tendency is presumed to have a more significant effect than the AT inhibition in severe acidemia (< pH 6.6), as confirmed in a preliminary study (data not shown) and the results of this study.

However, *in vivo* studies using a swine model (11, 12) confirmed the impairment of most coagulation parameters, even at pH 7.1. *In vitro* studies in which blood is acidified outside the patient are difficult to compare with studies in which acidemia is present in the patient. *In vitro* acidification does not consider the possible effects of acidemia on tissues or the *in vivo* interplay with the endothelium. Significant coagulopathy can only be induced when unrealistic levels of acidemia are induced *in vitro*, which are difficult to achieve *in vivo*.

Therefore, this study induced more severe acidemia than that induced in previous *in vitro* studies to discern the distinct impact of *in vitro* acidemia in dogs, considering the limited research and inconsistent results in assessing the effect of acidemia on coagulation in dogs. Additionally, the significant PT and aPTT prolongation in this experiment can be attributed to testing equipment differences. The coagulation analyzer employed in this investigation possessed a broader measurement range, which may have contributed to the observed significant statistical differences, unlike what was observed when the STA-compact analyzer was utilized in a previous study (15).

Ultimately, reversing acidemia reversed the coagulation impairment in this study. Significant recovery was confirmed for many coagulation parameters, and a trend towards recovery was observed for all parameters, except fibrinogen and TMA, without significant differences. These findings are consistent with previous *in vitro* experiments using human whole blood (16). However, an *in vivo* study using a swine model showed that Tris buffer infusion successfully corrects pH but does not significantly ameliorate coagulopathy (26). The experimental results appear conflicting, and to the best of our knowledge, no studies investigating whether acidemia-induced coagulopathy can be recovered by reversing acidemia in dogs have been conducted. Further investigations are necessary to determine whether pH correction can improve acidemia-induced coagulopathy *in vivo*.

A potential limitation of this study is the *in vitro* design. *In vitro* experiments are minimally invasive and offer high control over experimental conditions. However, they may lack physiological relevance because they do not fully reproduce the complex interplay of physiological systems in living organisms, limiting their application in clinical settings. Another limitation is the small number of enrolled dogs. Additional research involving a larger number of dogs is required to ascertain whether coagulation failure also occurs in dogs with clinical acidosis and whether it can be reversed by correcting acidemia *in vivo*.

In conclusion, the *in vitro* induction of acidemia in canine whole blood resulted in the progressive impairment of various coagulation parameters, including alterations in PT, aPTT, TEG parameters, and velocity curves. Reversing acidemia has the potential to recover acidemia-induced coagulation impairments *in vitro*. Therefore, coagulation tests should be conducted on dogs with acidemia in clinical practice to confirm whether coagulation failure occurs owing to acidemia, as demonstrated by *in vitro* experiments. Further research is required to determine whether reversing acidemia can improve coagulation dysfunction *in vivo*.

## Data Availability

The raw data supporting the conclusions of this article will be made available by the authors, without undue reservation.
